# Diagnostic Performance of ^18^F-FDG PET/CT in Native Valve Endocarditis: Systematic Review and Bivariate Meta-Analysis

**DOI:** 10.3390/diagnostics10100754

**Published:** 2020-09-25

**Authors:** Christel H. Kamani, Gilles Allenbach, Mario Jreige, Anna G. Pavon, Marie Meyer, Nathalie Testart, Maria Firsova, Victor Fernandes Vieira, Sarah Boughdad, Marie Nicod Lalonde, Niklaus Schaefer, Benoit Guery, Pierre Monney, John O. Prior, Giorgio Treglia

**Affiliations:** 1 Department of Nuclear Medicine and Molecular Imaging, Lausanne University Hospital, CH-1011 Lausanne, Switzerland; Christel-Hermann.Kamani@chuv.ch (C.H.K.); Gilles.Allenbach@chuv.ch (G.A.); Mario.Jreige@chuv.ch (M.J.); Marie-Madeleine.Meyer@chuv.ch (M.M.); Nathalie.Testart@chuv.ch (N.T.); Maria.Firsova@chuv.ch (M.F.); Victor.Fernandes-Vieira@chuv.ch (V.F.V.); Sarah.Boughdad@chuv.ch (S.B.); Marie.Nicod-Lalonde@chuv.ch (M.N.L.); Niklaus.Schaefer@chuv.ch (N.S.); John.Prior@chuv.ch (J.O.P.); 2 Department of Cardiology, Lausanne University Hospital, CH-1011 Lausanne, Switzerland; Anna-Giulia.Pavon@chuv.ch (A.G.P.); Pierre.Monney@chuv.ch (P.M.); 3 University of Lausanne, CH-1011 Lausanne, Switzerland; Benoit.Guery@chuv.ch; 4 Department of Infectious Diseases, Lausanne University Hospital, CH-1011 Lausanne, Switzerland; 5 Clinic of Nuclear Medicine and Molecular Imaging, Imaging Institute of Southern Switzerland, Ente Ospedaliero Cantonale, CH-6500 Bellinzona, Switzerland; 6 Health Technology Assessment Unit, Academic Education, Research and Innovation Area, Ente Ospedaliero Cantonale, CH-6500 Bellinzona, Switzerland

**Keywords:** diagnostic performance, ^18^F-FDG, PET/CT, positron emission tomography, native valve endocarditis, infectious endocarditis, infectious diseases, meta-analysis, systematic review

## Abstract

Background: Infectious endocarditis is a life-threatening disease, requiring prompt and accurate diagnosis. The aim of this article is to perform a systematic review and meta-analysis of the literature to estimate the performance of fluorine-18 fluorodeoxyglucose positron emission tomography/computed tomography (^18^F-FDG PET/CT) for the diagnosis of native valve endocarditis (NVE). Methods: Selected articles evaluating the diagnostic accuracy of ^18^F-FDG PET/CT in patients with suspected NVE, resulting from a comprehensive literature search through the PubMed/MEDLINE and Cochrane library databases until April 2020, were included for the systematic review and meta-analysis. Results: Seven studies (351 episodes of suspected NVE) were included. ^18^F-FDG PET/CT yielded a pooled sensitivity of 36.3% and a pooled specificity of 99.1% for the diagnosis of NVE. The pooled positive likelihood ratio, negative likelihood ratio, and diagnostic odds ratio were 8.3, 0.6, and 15.3, respectively. The sensitivity increased using contemporary PET/CT device with state-of-the-art patient preparation as well as innovative image acquisitions or adding the results of ^18^F-FDG PET/CT in a multimodality strategy. Conclusions: In our systematic review and meta-analysis, ^18^F-FDG PET/CT yielded a poor pooled sensitivity with an otherwise excellent pooled specificity for the diagnosis of NVE; however, several factors may increase the sensitivity without affecting the specificity and these factors should be better evaluated in future studies.

## 1. Introduction

Infective endocarditis (IE) is a multisystem infectious disease affecting the damaged endocardial surface of the heart valve as well as the surface of an indwelling cardiac device [1]. It was first described as a pathological infectious process in 1885 by Sir William Osler [2]. It is a relatively rare disease, with a prevalence ranging from 1.5 to 11.6 cases per 100,000 person-years [3]. IE occurs in more than half of cases in the native valve [4] and it remains a life-threatening disease despite the improvement in diagnosis and therapy, with contemporary mortality rates up to 25% [5]. The diagnosis of native valve endocarditis (NVE) is based on the presence of positive blood cultures and evidence of endocardial involvement in echocardiography as major criteria [6]. However, using the Duke criteria (DC) alone leads to about 24% misclassification of patients with proven IE to possible IE, especially by negative blood culture endocarditis [7]. This is of clinical importance, since delay in the diagnosis and thus initiation of an appropriate treatment results in poorer clinical outcome [8]. Therefore, further strategies for prompt and accurate diagnosis are still needed.

In recent years, promising studies on the role of fluorine-18 fluorodeoxyglucose positron emission tomography/computed tomography (^18^F-FDG PET/CT) in the diagnosis of active inflammatory and infectious processes in patients with fever of unknown origin, with endovascular prosthesis and implantable pacemaker/defibrillators have been published [9]. Moreover, the potential of this technique to diagnose prosthetic valve endocarditis (PVE) [10] has also been investigated. This investigation tool relies on the increase glycolytic activity of inflammatory cells following pathogen invasion, allowing their visualization by ^18^F-FDG PET/CT [11]. Based on accumulating evidence, ^18^F-FDG PET/CT has been included as a new major criterion for PVE in the modified Duke criteria (mDC) endorsed by the European Society of Cardiology in 2015 [6]. However, the evidence is not in favor of the use of this technique for the diagnosis of NVE due to its limited sensitivity [6].

New evidence on the value of ^18^F-FDG PET/CT in IE has been published in over the last few years. Some of these studies were pooled in three systematic reviews and/or meta-analyses with mixed population of IE patients [12,13,14]. We perform this systematic review and meta-analysis to investigate the current value of ^18^F-FDG PET/CT in the detection of NVE only.

## 2. Materials and Methods

The authors performed a systematic review and meta-analysis according to the “Preferred Reporting Items for a Systematic Review and Meta-Analysis of Diagnostic Test Accuracy Studies” (PRISMA-DTA statement), a guideline which describes the items required for reporting in systematic reviews and meta-analyses of diagnostic accuracy studies [15].

### 2.1. Search Strategy

We performed a comprehensive computer literature search through PubMed/MEDLINE and Cochrane library databases for published studies evaluating the diagnostic accuracy of ^18^F-FDG PET/CT in patients with suspected NVE until April 27, 2020. For this purpose, a search algorithm based on a combination of terms was created and used: (A) “PET” OR “positron” OR “FDG” AND (B) “endocarditis”. There were no date limit nor language restrictions applied to the database search. Moreover, in order to achieve a more comprehensive search, we also manually screened the references of the selected articles.

### 2.2. Study Selection

Titles and abstracts of the records obtained by using our search strategy were independently screened by two reviewers (C.K. and G.T.) based on predefined inclusion and exclusion criteria. Inclusion criteria were original articles reporting information on the diagnostic performance of ^18^F-FDG PET/CT in NVE. Exclusion criteria were: (a) reviews, editorials, comments related to the review question; (b) case reports related to the review question; (c) articles outside the field of interest of this review (including those focused on the role of ^18^F-FDG PET/CT in detecting PVE or extra-cardiac foci of IE). The full text of letters and original articles which were related to the review question was independently screened to assess for their inclusion for both the systematic review and the meta-analysis. From these selected articles, those who did not provide sufficient data to reassess the sensitivity and specificity of ^18^F-FDG PET/CT in NVE were excluded from the meta-analysis. In the case of data overlap, all the selected articles were included in the systematic review, whereas only the article with the most complete information was included in the meta-analysis. Disagreements were solved through a consensus meeting between both reviewers.

### 2.3. Data Extraction

For each included study, data were extracted by two reviewers (C.K. and G.T.), including general information on study characteristics (authors name, year of publication, country, study design), patient characteristics (age, sex ratio, type and number of patients evaluated, clinical and biological findings), technical details (patient preparation protocol, ^18^F-FDG injected activity, time interval between radiotracer injection and image acquisition, acquisition of delayed images, and protocol for the image analysis). Moreover, data on the diagnostic accuracy of ^18^F-FDG PET/CT on a per patient-based analysis (including true positive and true negative findings, false positive and false negative findings, sensitivity, specificity, positive and negative predictive values, diagnostic accuracy), name of the most common microbiological pathogen involved in NVE and information about the extent of the infection were also collected. For some articles, extraction of data from available figures, followed by mathematic calculations, was needed to calculate the diagnostic value of ^18^F-FDG PET/CT.

### 2.4. Quality Assessment

The quality of the included studies was assessed according to the revised “Quality Assessment of Diagnostic Accuracy Studies” tool (QUADAS-2) by 2 independent investigators (C.K. and G.T.). Of note, QUADAS-2 includes four domains: patient selection, index test, reference standard, and flow and timing. Each domain was assessed in terms of risk of bias, and also in terms of concerns regarding applicability (except the flow and timing domain for this latest) [16]. Any discrepancies in the quality assessment were solved by consensus.

### 2.5. Statistical Analysis

On a per patient-based analysis, accuracy data (true positive, false positive, true negative, and false negative) were extracted from each study to assess the pooled sensitivity and specificity, using a bivariate random-effects model for statistical pooling of data, which takes into account any possible correlation between sensitivity and specificity. Further diagnostic measures were calculated, including positive and negative likelihood ratios (LR+ and LR–) as well as diagnostic odds ratio (DOR). A random-effects model was also used for statistical pooling of LR+, LR–, as well as DOR. Pooled data were given with 95% confidence intervals (95% CI) and displayed using forest plots. Heterogeneity was assessed using the I-square index (I^2^). Statistical analyses were performed using OpenMeta [Analyst]^®^ software (version 0.1503) funded by the Agency for Healthcare Research and Quality (AHRQ) (Rockville, Maryland, United States).

## 3. Results

### 3.1. Literature Search

**Figure 1:**
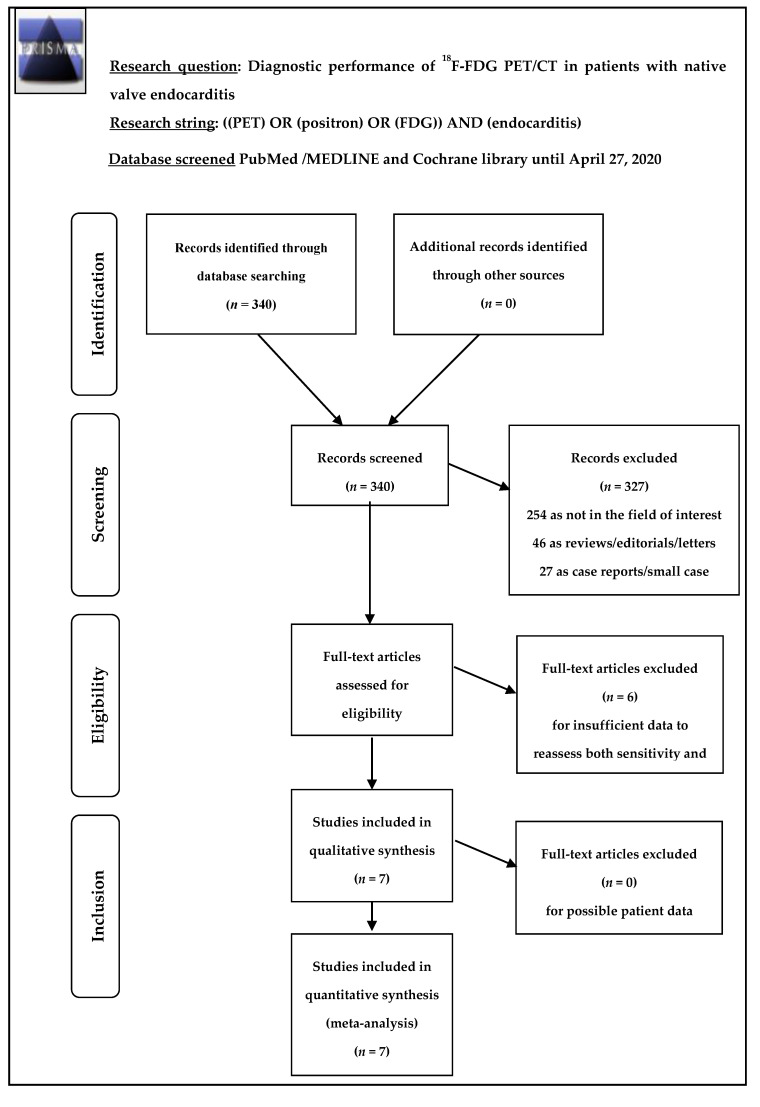
Flowchart of study selection and search results.

A total of 340 records were identified through the comprehensive electronic database search of PubMed/MEDLINE and Cochrane library databases. Overall, 327 records were excluded: 254 as not in the field of interest, 46 as reviews, editorials, or letters, and 27 as case reports. Thus, 13 articles were eligible for full-text screening. No pertinent studies were added after screening the references of these articles. After full-text screening, six articles were excluded from the systematic review as well as from the meta-analysis, because of missing information to assess both sensitivity and specificity of ^18^F-FDG PET/CT. Therefore, seven articles with a total of 351 episodes of suspected NVE were included in the qualitative (systematic review) as well as in the quantitative analysis (meta-analysis) [17,18,19,20,21,22,23]. Figure 1 summarizes the literature search results.

### 3.2. Qualitative Analysis (Systematic Review)

#### 3.2.1. Basic Study and Patient Characteristics

Table 1a,b summarize the main characteristics of the included studies. Of note, the selected studies were mostly performed in Europe, except for two which were performed in North America [17] and South America [19]. There were all monocentric and mostly prospective, except for two retrospective studies [17,22]. The articles were published between 2016 and 2020. Five studies included a total of 722 episodes with clinical suspicion of IE, with a subgroup of 209 episodes with suspected NVE [18,19,20,21,23]. Two studies were exclusively dedicated to assess the accuracy of ^18^F-FDG PET/CT in 142 patients with clinical suspected NVE [17,22]. In six studies, the mean age of the patients ranged from 58 to 71 years with a proportion of 68.1% of males, whereas corresponding data could not be assessed in one study [23]. Two studies reported the percentage of positive echocardiographic findings, ranging from 19% to 42% [20,22]. These two studies reported a rate of positive blood culture of 52% and 100% of the study population, partly because only blood culture positive patients were included in the second study by Kouijzer et al. [22]. A third study reported a 74% rate of positive blood culture [19]. Two studies reported the presence of fever in about 70% of their study population [17,19], with a C-reactive protein (CRP) mean value of 78.5 mg/L in one of these studies [17], similar to the CRP mean value of another study [18]. Most patients were treated with antibiotics prior to ^18^F-FDG PET/CT, with a mean duration of treatment ranging from 7 to 30 days [17,19,20,21].

#### 3.2.2. Technical Aspects

Table 2a,b summarize the technical aspects of ^18^F-FDG PET/CT in the included studies. In all studies, the physiological myocardial ^18^F-FDG uptake suppression was obtained through high-fat low-carbohydrate (HFLC) diet for 12 to 24 h and at least 6 to 8 h prolonged fasting prior administration of ^18^F-FDG. Moreover, a bolus of unfractionated heparin (50 IU/kg) was injected, when not contraindicated [17,18,21]. No indication about heparin administration was given in four studies [19,20,22,23]. The images were acquired 60 min after administration of ^18^F-FDG in the vast majority of studies, except for one, where the images were performed earlier at 45 min [18]. Four studies used a Biograph mCT hybrid PET/CT (Siemens) [17,20,21,22] with different technical performance, and one used a Gemini-TF hybrid PET/CT (Philips) [19]. No information about the type of hybrid device was given in two studies [18,23]. The mean injected activity ranged from 3 to 5 MBq/kg in four studies [19,20,21,22]. A fixed dose of 370 MBq was administered in two studies [17,18]. The images were visually assessed in all studies by two experienced physicians, all blinded to the patient clinical information. In two studies, involvement of a third experienced physician was required in case of disagreement [18,21]. For the vast majority of studies, a focal or heterogeneous ^18^F-FDG uptake greater than the surrounding tissue, after exclusion of potential residual physiological myocardial activity, was used as positive criterion [17,19,20,21,23]. Two studies considered any increased ^18^F-FDG uptake in the native heart valve as a positive criterion [18,22]. In addition, visual assessment was standardized using a scoring system in two studies [20,21]. Three studies used in addition to the visual analysis a semi-quantitative assessment of the ^18^F-FDG uptake, i.e., through the calculation of the maximal standardized uptake values (SUVmax) [18,19,21].

#### 3.2.3. Main Findings

**Figure 2:**
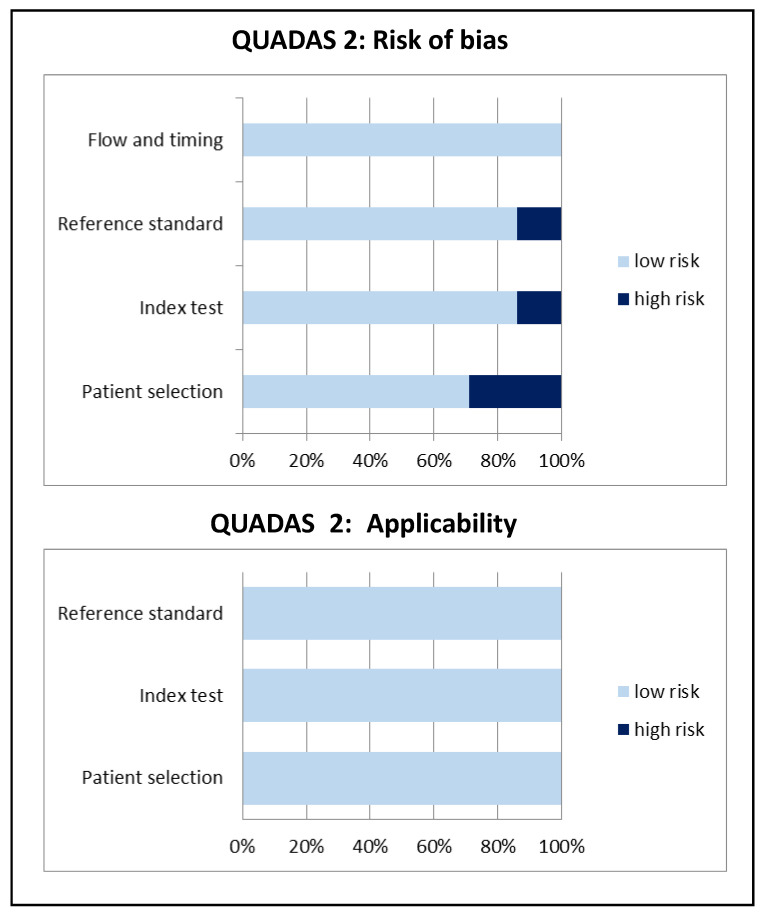
Overall quality assessment of the studies included in the systematic review according to Quality Assessment of Diagnostic Accuracy Studies (QUADAS-2) tool.

Table 3a,b summarize the diagnostic performance of ^18^F-FDG PET/CT in patients suspected of NVE in the included studies, whereas Figure 2 illustrates the overall quality assessment of these studies. No adverse effects were reported in any of the patients after ^18^F-FDG administration. In most of the included studies, we observed a poor sensitivity of ^18^F-FDG PET/CT in patients with suspected NVE, except for one recent study with improved sensitivity by 67.7% [17]. In contrast, the specificity of ^18^F-FDG PET/CT was excellent in all included studies, reaching 100% in four studies. The diagnostic accuracy ranged from 61.8% to 85.2%.

The most common microbiological pathogens found in blood cultures were Gram-positive bacteria from the Staphylococcus species, followed by Streptococcus species and Enterococcus species. Due to the acquisition from the base of the skull to at least the mid-tights in all the studies, it was possible to assess for the presence of metastatic foci. These were reported in the majority of studies in patients with confirmed NVE, in up to 54.5% of the patients [22]. Alternative diagnoses, such as systemic inflammatory diseases or neoplastic diseases, were reported in the majority of studies in patients with rejected diagnosis of NVE, in up to 45% of the patients [21]. These studies, except one [23], also reported alternative foci of infections. It should be mentioned that the exact percentage of metastatic foci and alternative diagnosis in patients with confirmed or rejected diagnosis of NVE could not be extracted for all the included studies, since these data were given for the mixed population of PVE and NVE rather than specifically for the NVE population. All studies used, as gold standard for the diagnosis of IE, the decision of a multidisciplinary endocarditis team (MET), applying the mDC while integrating clinical, imaging, and microbiological findings. Three studies used, additionally in the final decision, the histological analyses of the valve tissue obtained after surgery [17,19,23]. Three studies excluded the ^18^F-FDG PET/CT findings from the final decision [17,18,19], whereas four studies integrated them in the final decision [20,21,22,23].

Two from seven studies [22,23] reported false positive cases. False negative cases were reported in all studies, ranging from 32% to even 100%; this latest in a study with limited number of patients [21]. One study reported incomplete ^18^F-FDG myocardial suppression as a potential cause of the false negative findings [17]. Two studies did not report any significant difference in the performance of ^18^F-FDG PET/CT between different groups based on the duration of antibiotic treatment [19,20], whereas one study reported the administration of antibiotics in the vast majority of patients with false negative results [17].

Echocardiography performed globally better than ^18^F-FDG PET/CT in the majority of studies [18,21,23]. Two studies reported a better performance of ^18^F-FDG PET/CT compared to the mDC [17,22]. However, in one study, only transthoracic echocardiography (TTE) and not transesophageal echocardiography (TEE) was performed in the majority of patients with the diagnosis of possible or rejected NVE [22]. Two studies reported a greater size of the vegetation in patients with positive ^18^F-FDG PET/CT findings [17,23]. When adding ^18^F-FDG PET/CT as major and minor criterion in the mDC, we observed a significant improvement of the mDC sensitivity up to 78% (95%CI 66–90%), without excellent specificity by up to 91% (95%CI 85–98), as reported in two studies [17,19].

### 3.3. Quantitative Analysis (Meta-Analysis)

**Figure 3:**
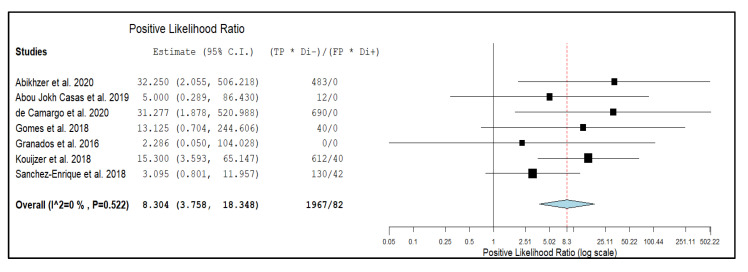
Forest plot of individual studies and pooled positive likelihood ratio of ^18^F-FDG PET/CT in patients with suspicious native valve endocarditis (NVE), including 95% confidence interval (95% CI). The size of the squares indicates the weight of each study. The blue rhombus indicates the pooled estimate with 95% confidence intervals.

**Figure 4:**
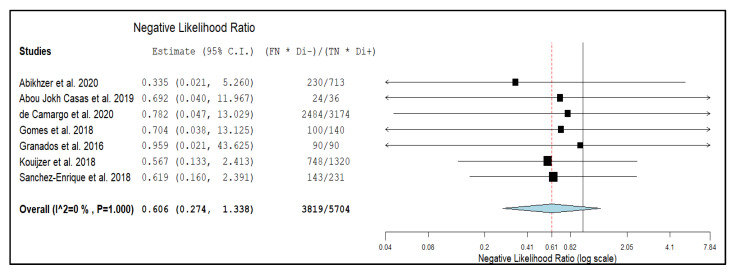
Forest plot of individual studies and pooled negative likelihood ratio of ^18^F-FDG PET/CT in patients with suspicious NVE, including 95% confidence interval (95%CI). The size of the squares indicates the weight of each study. The blue rhombus indicates the pooled estimate with 95% confidence intervals.

**Figure 5:**
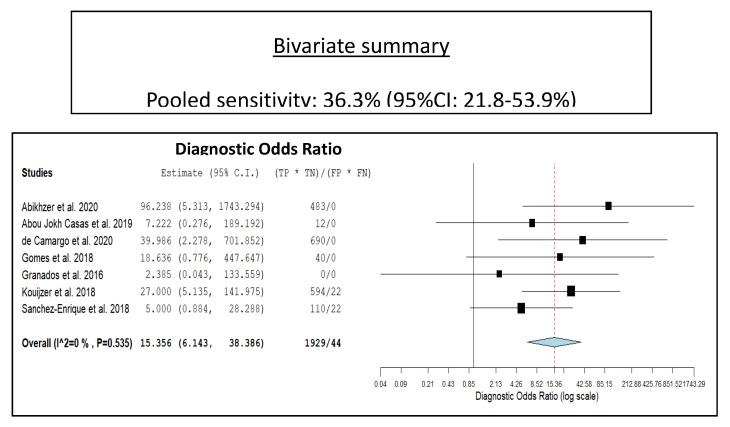
Forest plot of individual studies and pooled diagnostic odds ratio of ^18^F-FDG PET/CT in patients with suspicious NVE, including 95% confidence interval (95%CI). The size of the squares indicates the weight of each study. The blue rhombus indicates the pooled estimate with 95% confidence intervals.

Seven studies with 351 episodes of suspected NVE were included for the bivariate meta-analysis [17,18,19,20,21,22,23]. Results of the meta-analysis are presented in Figure 3, Figure 4, and Figure 5. The sensitivity of ^18^F-FDG PET/CT in patients with suspected NVE ranged from 22% to 67.7%, with a pooled estimate of 36.3% (95%CI: 21.8–53.9%). The specificity of ^18^F-FDG PET/CT in patients with suspected NVE ranged from 84.6% to 100% with a pooled estimate of 99.1% (95%CI: 88.6–99.9%). The pooled LR+, LR−, and DOR were 8.3 (95%CI: 3.7–18.3), 0.6 (95%CI: 0.27–1.33), and 15.3 (95%CI: 6.14–38.38), respectively. No significant statistical heterogeneity among the studies was found for all the metrics evaluated (*I*^2^ = 0%).

## 4. Discussion

Three recent systematic reviews and/or meta-analyses have questioned the performance of ^18^F-FDG PET/CT in the diagnosis of IE [12,13,14]. In recent years, several articles assessing the diagnostic performance of ^18^F-FDG PET/CT in NVE became available. To better understand the potential role of ^18^F-FDG PET/CT in the diagnosis of NVE, we pooled the results from several studies to increase the statistical power.

In this meta-analysis of seven mostly prospective studies including 351 episodes with suspected NVE, ^18^F-FDG PET/CT showed a poor pooled sensitivity (36.3%) but an excellent pooled specificity (99.1%) for the diagnosis of NVE. Moreover, whole body ^18^F-FDG PET/CT was able to assess the extent of the infection, being the first modality to diagnose extra-cardiac foci in up to 79% of cases [22]. ^18^F-FDG PET/CT was also able to provide alternative infectious foci by rejected NVE diagnosis as well as noninfectious relevant diagnosis, such as neoplastic or inflammatory disease [17,18,19,20,21,22].

In four studies, ^18^F-FDG PET/CT findings were included in the final decision of the MET [20,21,22,23]. This could have led to an underestimation of the false positive cases, and thus contribute to the excellent specificity. However, three others studies [17,18,19] excluded the ^18^F-FDG PET/CT results from the final decision, always with an excellent specificity by 100%. Moreover, two of these studies had an external validation using histopathological findings, whenever available. These observations confirm the excellent specificity of ^18^F-FDG PET/CT for this indication. This specificity is clearly superior in this indication in comparison to PVE [12,13]. The reason for this superiority is the absence of prosthetic material that can cause reactive post-operative ^18^F-FDG uptake.

Heterogeneity in patient preparation has contributed to the poor sensitivity of ^18^F-FDG PET/CT in the diagnosis of NVE. Due to the physiological myocardial glucose uptake, a metabolic shift of the myocardium exclusively towards fatty acid metabolism is required before administration of ^18^F-FDG, an analog of glucose. This can be achieved using different techniques, including dietary manipulation, as well as administration of some drugs such as heparin, known to increase the lipolysis [24]. The only study not including a HFLC diet in the patient preparation reported a relatively low rate of complete myocardial glucose uptake suppression (61% of patients with suspected PVE and NVE) and the poorest sensitivity (0%) of the included articles [21]. Some studies decided to exclude patients with inadequate ^18^F-FDG myocardial suppression [19,22], introducing a selection bias in their results. This inadequate patient preparation is likely the reason for the false positive cases reported in two studies, since no physiological valvular or paravalvular ^18^F-FDG uptake is expected, except from the surrounding myocardium [22,23]. Abikhzer et al. [17] reported incomplete ^18^F-FDG myocardial suppression in 60% of the false negative studies. Interestingly, after excluding these patients, the ^18^F-FDG PET/CT sensitivity significantly increased from 67.7% to 80%, without altering the excellent specificity. This sensitivity remains lower than the reported high sensitivity of TEE for NVE, in the range of 95%–100% [25]. However, based on its higher specificity, ^18^F-FDG PET/CT could be considered in patients with possible NVE after doubtful or negative TTE or with a contra-indication for TEE [17,22]. Since the scans results have major implications on patient management, it is of the utmost importance to implement standardized preparation protocols with higher rate of adequate ^18^F-FDG myocardial suppression [26].

Inflammatory cells, especially monocytes, are constituents of the inflammatory response following adhesion and invasion of the valvular site by pathogens, with a marked increase of their glycolytic activity, allowing their visualization by ^18^F-FDG PET/CT [11]. In 54 patients with NVE, De Carmargo et al. [19] compared the ^18^F-FDG uptake pattern with histopathological analysis, including the presence of fibrin, inflammatory infiltrate, and fibrosis. They demonstrated a significantly lower accumulation of fibrin, inflammatory cells, especially polymorphonuclear cells, and a significant higher content of fibrosis in NVE samples in comparison to PVE samples. These authors associated the reduced accumulation of inflammatory cells in NVE with the fact that vegetations are avascular and mostly of small size (less than 10 mm). The direct consequence is the reduced ^18^F-FDG uptake by NVE, insufficient to be clearly differentiated from the background activity, and thus to be visualized by ^18^F-FDG PET/CT. Moreover, the constant movement of the valve makes the situation worse since the already low ^18^F-FDG signal is being blurred by this valve’s motion [6]. In one study, the use of innovative acquisition sequence, such as delayed acquisitions (134 ± 42 min post injection of ^18^F-FDG, compared to the standard acquisition performed 45-60 min post injection of ^18^F-FDG) was shown to contribute to a significant increase the ^18^F-FDG PET/CT performance to diagnose NVE [17]. Indeed, the authors demonstrated an increase of the target to background ratio (TBR) on delayed images in comparison to standard images, allowing the identification of a large number of positive findings. Moreover, in this study, acquisitions of simultaneous electrocardiogram- and respiratory-gated cardiac images with motion correction provided a better contrast resolution (more counts per pixel), a better spatial resolution (larger matrix and smaller pixel size), as well as less scattering, leading to an improved sensitivity to detect lower metabolic activity, as well as better metabolic and anatomic image fusion [17]. This evidence points out the technical differences of the PET/CT systems, and explains the superiority in the detection rate of NVE in the one recent study using a contemporary PET/CT device [17].

Since the diagnosis of NVE is challenging, this meta-analysis emphasizes the need for a MET to include as much clinical and imaging information as possible in the final diagnosis. Four studies reported the performance of mDC in the detection of NVE as compared to ^18^F-FDG PET/CT. In two of them, ^18^F-FDG PET/CT was superior to mDC: in one of these studies [22], mostly TTE rather than TEE was performed in patients with possible or rejected diagnosis of NVE, whereas in the other one [17], use of delayed PET/CT acquisitions as well as a contemporary PET/CT device allowed for the diagnosis of most of the positive cases. In the two other studies, mDC was superior to ^18^F-FDG PET/CT [19,21]. Thus, compared individually to mDC, ^18^F-FDG PET/CT showed variable performance. Two studies [17,19] proposed new criteria of the mDC based on the ^18^F-FDG PET/CT findings and assessed their additive value on the overall performance of mDC. The major criterion was defined as a focal valvular or paravalvular ^18^F-FDG uptake. The minor criterion was defined as the presence of septic emboli. Indeed, ^18^F-FDG PET/CT as whole-body imaging method can detect septic emboli (except for the brain due to the increased cerebral glycolytic activity [11]), allowing the diagnosis of peripheral infectious complications. When integrating these new criteria in the mDC, its sensitivity increased up to 78%, without significantly affecting its specificity, which was up to 91% [17,19]. This led to a reclassification for up to 44% of patients from possible to definite NVE [17,19]. In the absence of an external gold standard, this performance could have been bias. Indeed, in this specific subgroup of patients with persistent suspicion of NVE and normal or doubtful echocardiography, no histological correlation between ^18^F-FDG uptake and the presence of infection can be made, since these patients usually had no indication for valve surgery. De Carmago et al. [19] made a histological correlation between ^18^F-FDG uptake and the histological confirmation of NVE in patients with confirmed NVE which undergone valve surgery. Thus, this evidence could be translated in this specific subgroup of patients, since no physiological ^18^F-FDG uptake is expected in the valvular area. However, the present data did not report on the number of patients with NVE with negative echocardiography, but positive valvular ^18^F-FDG uptake. This information would have allowed a better assessment of the valvular ^18^F-FDG uptake performance, since the value of septic emboli is still established in this specific group of patients [6]. Nevertheless, based on these promising results, in patients with doubtful echocardiography findings, the use of ^18^F-FDG PET/CT may be helpful in the final diagnosis of NVE [17,19]. Furthermore, ^18^F-FDG PET/CT in some cases revealed the presence of malignant lesions, alternative infectious foci, or other inflammatory diseases, such as cardiac sarcoidosis, with the possibility of an early curative management [17,18,19,20,21,22]. Gomes et al. [20] pointed out the additive value of the use of flowchart integrating multimodality imaging to increase the detection performance of NVE. Integrating the findings of ^18^F-FDG PET/CT with cardiac computed tomography angiography (CTA) and echocardiography (TTE and TEE) following a specific flowchart was feasible in clinical practice, providing complementary information on the anatomical cardiac structures and the extension of the infection. This led to significant increase in sensitivity for the diagnosis of NVE up to 86%, as compared to the sensitivity of each individual imaging modality. Finally, the specific patient referral through an MET increased the prevalence of the IE in the population referred for imaging investigation, leading to increased performance of the imaging tool. All this evidence could be discussed by the update of the next guidelines on the diagnosis of IE.

The suspicion of NVE requires the administration of large spectral antibiotics [6]. Since cardiac ^18^F-FDG PET/CT requires a dietary preparation, it is mostly performed a few days after introduction of an antibiotic treatment. Three studies reported on the impact of antibiotic treatment duration in the performance of ^18^F-FDG PET/CT [17,19,20]. In one study, the vast majority of patients with false negative results (9/10) were under antibiotic treatment [17]. Even if no information about the length of the treatment prior to PET/CT was provided, it can be suggested that antibiotics treatment could have led to false negative ^18^F-FDG PET/CT. This would be in line with observations in PVE. Indeed, a prolonged antibiotic therapy could bring the pathogens in a quiescent status, leading to less accumulation of the inflammatory cells at the site of infection and therefore less or absent ^18^F-FDG accumulation [27,28]. However, two other studies showed no significant difference on the performance of ^18^F-FDG PET/CT depending on the duration of the antibiotic treatment [19,20]. Of note, in these two studies, the sensitivity was much lower compared to the first study [17]. This controversy has to be further investigated in future studies.

The most common microbiological pathogens found as causes of NVE were Staphylococcus aureus (SA), followed by Streptococcus species (SS). The included studies did not perform cost-effectiveness analysis about the role of ^18^F-FDG PET/CT in NVE. Interestingly, a previous study performed a cost-effectiveness analysis of the use of ^18^F-FDG PET/CT in SA and SS bacteremia. This study demonstrated a decrease in morbidity and mortality when ^18^F-FDG PET/CT was included in the diagnostic algorithm to search for metastatic foci in patients with suspected endocarditis, with mean incremental costs within the range considered to be efficient by Dutch guidelines [29]. In this study, specific criteria to stratify the patient embolic risk were defined, such as persistent fever more than 72 h after administration of appropriate treatment, persistent positive blood culture over 48 h after administration of appropriate treatment, signs of infection 48 h prior administration of appropriate treatment, and community acquisition. The authors concluded that in patients with one or more of these risk features, there was a clinical, cost-effective benefit when ^18^F-FDG PET/CT was performed for the search of embolic foci.

Finally, no studies evaluating the accuracy of SUVmax could demonstrate any utility compared to the visual assessment. Therefore, this semi-quantitative score value is not recommended for the diagnosis of NVE in the clinical routine. This can be explained by the fact that many external factors (i.e., time interval between injection of ^18^F-FDG and image acquisition, plasma glucose, PET/CT scanner performances, etc.) can influence the SUVmax, resulting in wide variation among patients [18]. Thus, establishing a threshold to detect infection can be challenging.

In this meta-analysis, we focused on the diagnostic performance of ^18^F-FDG PET/CT for the detection of NVE. However, the diagnostic performance of an imaging test is not the only component of a successful clinical management. Other factors influence the clinical management of patients with suspected NVE, such as availability, local expertise in images interpretation, radiation dose, safety, examination time, organization, and cost-effectiveness.

In this meta-analysis, ^18^F-FDG PET/CT demonstrated poor pooled sensitivity and excellent specificity. However, many limiting factors were identified, which can be considered in future larger prospective and multicenter studies. Specifically, the use of standardized preparation protocol for ^18^F-FDG myocardial suppression with higher success rates should be implemented in further studies. Moreover, the value of semi-quantitative analysis of PET/CT images should be further assessed. Additional studies are also needed to evaluate the simultaneous acquisition of ^18^F-FDG PET/CT with cardiac CTA as well as the diagnostic accuracy of new PET/CT generation with higher timing and spatial resolution.

Some limitations and biases of our meta-analysis should be mentioned. First of all, a limited number of studies were available. Due to this limited number of studies, no significant biases among the included studies were identified. Heterogeneity among the studies (i.e., due to different characteristics among the included patients, difference in methodological aspects, and difference in gold standard) could also represent a potential source of bias in our meta-analysis [30]. On the other hand, we did not find a significant statistical heterogeneity among the studies included in our pooled analysis. The absence of a true independent gold standard could have introduced a certain bias in the evaluation of the ^18^F-FDG PET/CT performance to detect NVE. More, in the specific subgroup of patients with possible NVE, but normal or doubtful echocardiography, the added value of valvular ^18^F-FDG uptake needs to be better assessed.

## 5. Conclusions

In this meta-analysis specifically designed to assess the performance of ^18^F-FDG PET/CT in the diagnosis of NVE, we demonstrated the poor pooled sensitivity and excellent pooled specificity of ^18^F-FDG PET/CT in this setting. However, when using contemporary PET/CT device with innovative image acquisitions, the sensitivity increased, with still excellent specificity. Moreover, including ^18^F-FDG PET/CT findings in the mDC further increased the mDC sensitivity for the detection of NVE without affecting its specificity. Finally, when adding the results of ^18^F-FDG PET/CT in a multimodality strategy, the overall sensitivity for the detection of NVE can be improved. However, these findings need to be confirmed in larger prospective multicenter studies.

## Figures and Tables

**Table 1:** (**a**) Basic study and patient characteristics; (**b**) Basic study and patient characteristics.

(a)							
Authors	Year	Country	Study Design		Type of Patients Evaluated		No. of Episodes with Suspicion of IE (NVE) Undergoing ^18^F-FDG PET/CT
Abikhzer et al. [17]	2020	Canada	Retrospective monocentric		Patients with clinical suspicion of NVE		54 (54)
Abou Jokh Casas et al. [18]	2019	Spain	Prospective monocentric		Patients with clinical suspicion of IE, including a subgroup of patients with suspicion of NVE		43 (12)
de Camargo et al. [19]	2020	Brazil	Prospective monocentric		Patients with clinical suspicion of IE, including a subgroup of patients with suspicion of NVE		303 (115)
Gomes et al. [20]	2018	Netherlands	Prospective monocentric		Patients with clinical suspicion of IE, including a subgroup of patients with suspicion of NVE		176 (27)
Granados et al. [21]	2016	Spain	Prospective monocentric		Patients with clinical suspicion of IE, including a subgroup of patients with suspicion of NVE		80 (21)
Kouijzer et al. [22]	2018	Netherlands	Retrospective monocentric		Patients with clinical suspicion of NVE		88 (88)
Sánchez-Enrique et al. [23]	2018	Spain	Prospective monocentric		Patients with clinical suspicion of IE, including a subgroup of patients with suspicion of NVE		120 (34)
**(b)**							
**Authors**	**Age** **(Years)**	**%Male**	**Vegetation in TTE/TEE**	**Positive Blood Cultures**	**Presence of Fever**	**Value of CRP**	**Antibiotic Treatment Prior to the Exam (days)**
Abikhzer et al. [17]	Mean: 63 ± 15 Range: 33–93	64.8%	NR	NR	37/54 (68.5%)	78.5 ± 43.2 (0.2–193)	Median duration: 7 (range 1–61 days).
Abou Jokh Casas et al. [18]	Median: 71 Range: 25–88	88.4%	NR	NR	NR	76 ± 75 (0–349)	NR
de Camargo et al. [19]	Mean: 58 ± 17	58%	NR	40/54 (74%)	82/115 (71%)	NR	6 ± 8 (1–62)
Gomes et al. [20]	Mean: 64 Range: 18–95	60%	5/27 (19%)	14/27 (51.8%)	NR	NR	Yes, length not specified
Granados et al. [21]	Mean: 68 ± 13	81%	NR	NR	NR	NR	Definite IE: 20 (12–30) Possible IE: 13 (8–25)
Kouijzer et al. [22]	Mean: 61 Range: 17–90	56.8%	37/88 (42%)	88/88 (100%)	NR	NR	NR
Sánchez-Enrique et al. [23]	NR	NR	NR	NR	NR	NR	NR

Legend: ^18^F-FDG = Fluorine-18 Fluorodeoxyglucose; IE = infective endocarditis; NVE = native valve endocarditis; PET/CT = positron emission tomography/computed tomography. CRP = C-reactive protein; IE = infective endocarditis; TTE = transthoracic echocardiography; TEE = transesophageal echocardiography; NR = not reported.

**Table 2:** **(a**) Technical aspects of fluorine-18 fluorodeoxyglucose positron emission tomography/computed tomography (^18^F-FDG PET/CT) in the included studies; (**b**)Technical aspects of 18F-FDG PET/CT in the included studies.

(a)					
Authors	Hybrid Imaging Modality Name of the Device	Myocardial Suppression of ^18^F-FDG Uptake	Protocol for Myocardial Suppression of ^18^F-FDG Uptake	Injection of Heparin (50 UI/Kg) 15 min Prior Injection of ^18^F-FDG	Mean Injected Activity
Abikhzer et al. [17]	PET/CT (with low-dose CT) Biograph mCT Flow 40 with TrueV, Siemens	Yes	Prolonged fasting (at least 12 h), HFLC diet in the 24 h prior injection of ^18^F-FDG	Yes, when not CI (10 patients excluded)	370 MBq
Abou Jokh Casas et al. [18]	PET/CT (with low-dose CT) NR	Yes	Prolonged fasting (at least 6 h), HFLC diet at in the 12 h prior injection of ^18^F-FDG	Yes, when not CI	370 MBq
de Camargo et al. [19]	PET/CT (with low-dose CT) Gemini-TF 64-Slice, Philips Medical Systems	Yes	Prolonged fasting (at least 8 h), HFLC diet in the 24 h prior injection of ^18^F-FDG	NR	5 MBq/kg
Gomes et al. [20]	PET/CT (with low-dose CT) Biograph mCT 64-Slice, Siemens	Yes	Prolonged fasting (at least 6 h), HFLC diet in the 24 h prior injection of ^18^F-FDG	NR	244 MBq (3 MBq/Kg)
Granados et al. [21]	PET/CT (with low-dose CT) Biograph mCT 64-Slice, Siemems	Yes	Prolonged fasting (at least 12 h)	Yes, when not CI	4 MBq/kg
Kouijzer et al. [22]	PET/CT (with low-dose CT) Biograph mCT 40, Siemens	Yes	Prolonged fasting (at least 6 h), HFLC diet in the 24 h prior injection of ^18^F-FDG	NR	3.3 MBq/kg
Sánchez-Enrique et al. [23]	PET/CT (with low-dose CT) NR	NR	NR	NR	NR
**(b)**					
**Authors**	**Time Interval between Radiotracer Injection and Image Acquisition**	**Delayed PET/CT Imaging**	**Image Interpretation**	**Visual Criteria for Positive ^18^F-FDG PET/CT**	**Semi-Quantitative Analysis**
Abikhzer et al. [17]	60 min	in selected cases	2 experienced physicians, blinded to the patient clinical information	Presence of focally increased ^18^F-FDG uptake in and around the native heart valve, excluding the papillary muscle	No
Abou Jokh Casas et al. [18]	45 min	NR	2 experienced physicians, with involvement of a third physician by disagreement	Presence of any increased ^18^F-FDG uptake in the native heart valve,	SUV_max_
de Camargo et al. [19]	60 min	NR	2 experienced physicians, blinded to the patient clinical information	Presence of focal or heterogeneous ^18^F-FDG uptake that persisted in the non-corrected images	SUV_max_
Gomes et al. [20]	60 min	NR	2 experienced physicians, blinded to the patient clinical information	Focal/heterogeneous ^18^F-FDG uptake, at least greater than uptake in mediastinum	No
Granados et al. [21]	60 min	NR	2 experienced physicians, with involvement of a third physician by disagreement	Presence of focal or heterogeneous ^18^F-FDG uptake that persisted in the non-corrected images	SUV_max_
Kouijzer et al. [22]	60 min	NR	2 experienced physicians, blinded to the patient clinical information	Presence of any increased ^18^F-FDG uptake in and around the native heart valve, distinguishable from normal heart uptake	No
Sánchez-Enrique et al. [23]	NR	NR	NR	Presence of focal or heterogeneous ^18^F-FDG uptake that persisted in the non-corrected images	No

Legend: ^18^F-FDG = Fluorine-18 Fluorodeoxyglucose; CI = contra-indicated; CT = computed tomography; HFLC = high-fat low-carbohydrate; MBq/kg = Mega Becquerel per kilogram; NR = not reported; PET/CT = positron emission tomography/computed tomography; min = minutes; mDC = modified Duke criteria; NR = not reported; PET/CT = positron emission tomography/computed tomography; SUVmax = maximal standardized uptake value.

**Table 3:** (**a**): Diagnostic performance of ^18^F-FDG PET/CT in patients with suspected native valve endocarditis; (**b**) Diagnostic performance of 18F-FDG PET/CT in patients with suspected native valve endocarditis.

(a)									
Authors.	Reference Standard for Diagnostic Performance Assessment			True Positive	False Positive	True Negative	False Negative	Sensitivity	Specificity
Abikhzer et al. [17]	histological analysis of valve tissue or mDC and decision of MET taking into account clinical, imaging, and microbiological findings. ^18^F-FDG PET/CT excluded from the final decision			21	0	23	10	67.7%	100%
Abou Jokh Casas et al. [18]	mDC determined by MET. ^18^F-FDG PET/CT excluded from the final decision			2	0	6	4	33.3%	100%
de Camargo et al. [19]	microbiological and histological analysis of valve tissue or modified Duke criteria determined by MET. ^18^F-FDG PET/CT excluded from the final decision			10	0	69	36	22%	100%
Gomes et al. [20]	mDC and decision of MET taking into account clinical, imaging, and microbiological findings. ^18^F-FDG PET/CT included in the final decision			2	0	20	5	28.6%	100%
Granados et al. [21]	mDC and decision of MET taking into account clinical, imaging, and microbiological findings. ^18^F-FDG PET/CT included in the final decision			0	0	15	6	0%	100%
Kouijzer et al. [22]	mDC and decision of MET taking into account clinical, imaging, and microbiological findings. ^18^F-FDG PET/CT included in the final decision			9	2	66	11	45%	97.1%
Sánchez-Enrique et al. [23]	histological analysis of valve tissue or mDC and decision of MET taking into account clinical, imaging, and microbiological findings. ^18^F-FDG PET/CT included in the final decision			10	2	11	11	47.6%	84.6%
**(b)**									
**Authors**	**PPV**	**NPV**	**Diagnostic Accuracy**	**Metastatic Foci**		**Alternative Diagnosis/ Foci for Infection**		**Most Common Microbiological Findings**	
Abikhzer et al. [17]	100%	69.7%	81.5%	45.1%		5 (21.7%)		NR	
Abou Jokh Casas et al. [18]	100%	60%	66.7%	NR		NR		1. Staph. epidermidis/ 2. Staph. aureus / 3. Strepto. bovis	
de Camargo et al. [19]	100%	66%	68.7%	Identified in 47 patients with final diagnosis, but not specifically reported for NVE cases		Identified in 29 patients with excluded IE, but not specifically reported for NVE cases		1. Strepto. sp. / 2. Staph. aureus / 3. Entero. sp.	
Gomes et al. [20]	100%	80%	81.5%	Identified in 6 patients with final diagnosis, but not specifically reported for NVE cases		Identified in 94% of patients with excluded IE, but not specifically reported for NVE cases		1. Staph. aureus / 2. Staph. others groups / 3. T. Whippeli	
Granados et al. [21]	NR	71.4%	71.4%	Identified in 8 patients with final diagnosis, but not specifically reported for NVE cases		7 (45%)		Staph. aureus	
Kouijzer et al. [22]	81.8%	85.7%	85.2%	Identified in 54.5% of patients with final diagnosis		NR		1. Staph. aureus / 2. Strepto. sp. / 3. Entero. sp.	
Sánchez-Enrique et al. [23]	83.3%	50%	61.8%	NR		NR		NR	

Legend: ^18^F-FDG PET/CT = fluorine-18 fluorodeoxyglucose positron emission tomography/computed tomography; mDC = modified Duke criteria; MET = multidisciplinary endocarditis team; Entero = Enterococcus; IE = infectious endocarditis; NPV = negative predictive value; NR = not reported; NVE = native valve endocarditis; PPV = positive predictive value; sp. = species; Staph = Staphylococcus; Strepto= Streptococcus; T = Tropheryma.
